# *HLA-B* and *C* Expression Contributes to COVID-19 Disease Severity within a South African Cohort

**DOI:** 10.3390/genes15040522

**Published:** 2024-04-22

**Authors:** Lisa Naidoo, Thilona Arumugam, Veron Ramsuran

**Affiliations:** 1 School of Laboratory Medicine and Medical Sciences, College of Health Sciences, University of KwaZulu-Natal, Durban 4041, South Africa; lisa08naidoo@gmail.com (L.N.); cyboglona@gmail.com (T.A.); 2 Centre for the AIDS Programme of Research in South Africa (CAPRISA), Nelson R. Mandela School of Medicine, University of KwaZulu-Natal, Durban 4041, South Africa

**Keywords:** COVID-19, *HLA-B*, *HLA-C*, disease severity

## Abstract

Globally, SARS-CoV-2 has negatively impacted many lives and industries due to its rapid spread, severe outcomes, and the need for the implementation of lockdown strategies across the world. SARS-CoV-2 disease severity varies among different populations. Host genetics have been associated with various diseases, and their ability to alter disease susceptibility and severity. In addition, Human Leukocyte Antigen (*HLA*) expression levels and alleles vary significantly among ethnic groups, which might impact the host’s response to SARS-CoV-2. Our previous study highlighted that *HLA-A* might have an effect on COVID-19 disease severity across ethnicities. Therefore, in this study, we aim to examine the effect of *HLA-B* and *C* expression levels on COVID-19 disease severity. To achieve this, we used real-time PCR to measure the *HLA* mRNA expression levels of SARS-CoV-2-infected individuals from a South African cohort and compared them across ethnic groups, disease outcomes, gender, comorbidities, and age. Our results show (1) that the effect of *HLA-B* mRNA expression levels was associated with differences in disease severity when we compare symptomatic vs. asymptomatic (*p* < 0.0001). While *HLA-C* mRNA expression levels were not associated with COVID-19 disease severity. (2) In addition, we observed that *HLA-B* and *HLA-C* mRNA expression levels were significantly different between South African Black individuals and South African Indian individuals (*p* < 0.0001, *p* < 0.0001). *HLA-B* mRNA expression levels among symptomatic South African Black individuals were significantly higher than symptomatic South African Indian individuals (*p* < 0.0001). In addition, the *HLA-B* mRNA expression levels of symptomatic South African Black individuals were significantly higher than asymptomatic South African Black individuals (*p* > 0.0001). *HLA-C* mRNA expression levels among symptomatic South African Black individuals were significantly higher than among symptomatic South African Indian individuals (*p* = 0.0217). (3) *HLA-C* expression levels were significantly different between males and females (*p* = 0.0052). In addition, the *HLA-C* expression levels of asymptomatic males are higher than asymptomatic females (*p* = 0.0375). (4) *HLA-B* expression levels were significantly different between individuals with and without comorbidities (*p* = 0.0009). In addition, we observed a significant difference between individuals with no comorbidities and non-communicable diseases (*p* = 0.0034), in particular, hypertension (*p* = 0.0487). (5) *HLA-B* expression levels were significantly different between individuals between 26–35 and 56–65 years (*p* = 0.0380). Our work is expected to strengthen the understanding of the relationship between *HLA* and COVID-19 by providing insights into *HLA-B* and *C* expression levels across ethnic populations in South Africa among COVID-19-symptomatic and asymptomatic individuals. Our results highlight that *HLA-B* mRNA expression levels contribute to COVID-19 severity as well as variation in ethnicities associated with COVID-19. Further studies are needed to examine the effect of *HLA* expression levels across various ethnic groups with contributing factors.

## 1. Introduction

COVID-19, a serious health burden, has severely and quickly disrupted the world in various ways [1]. SARS-CoV-2, the cause of COVID-19, will continue to mutate and escape the human immune response and human therapeutics for years to come until serious innovations to eliminate the virus are implemented. Since viral genetics change rapidly, it would be more beneficial if we focused our attention on host genetics. More research on host genetic factors and their role in SARS-CoV2 susceptibility and disease progression is required. COVID-19 severity rates have been associated with viral mutations [2]. COVID-19 has been associated with a range of symptoms, from asymptomatic and mildly symptomatic to severe infection and death [3,4]. The varying severity experienced during COVID-19 could be attributed to host genetics. Africa was not as severely affected due to contributing factors such as other infectious diseases, co-morbidities, age, and genetics. Elderly individuals and individuals with comorbidities are at risk for severe COVID-19 [1]. Identifying host genes associated with these contributing factors and lowering COVID-19 severity among the African population is imperative. The disparities in COVID-19 outcomes are multifactorial. Therefore, it is important to include these factors in research.

Host genetics has been shown to have a significant impact on disease outcomes [5,6]. The *HLA* system is the first line of defense, and it is imperative for the identification of foreign particles and the immune response. *HLA* class I molecules (*HLA-A*, *-B*, and *C*) bind and present peptides from intracellular proteins to cytotoxic CD8+ T cells. An effective immune response is dependent on the proper presentation of the viral peptides on the host cell surface, along with other factors [7,8,9,10]. Other infectious diseases and disorders have been associated with *HLA*. *HLA* polymorphisms have impacted the severity of other RNA viruses, like SARS-CoV-2 [1,11]. Therefore, other studies have analyzed *HLA* and shown the growing evidence of its importance in COVID-19 pathogenesis [12,13,14]. Varying *HLA* alleles and expression levels may lead to different peptide presentations, which may lead to different individuals having varying COVID-19 susceptibility and severity. *HLA* has been suggested as a potential genetic host factor that influences individuals immune responses to SARS-CoV-2. *HLA-A* and *HLA-B* are expressed higher than *HLA-C*. *HLA-A* and *HLA-B* are found on nucleated cells, while *HLA-C* is found on lymphocytes and dendritic cells. These molecules are expressed differently and at different levels. *HLA-C, HLA-A,* and *HLA-B* are highly polymorphic, but these levels might differ between them. *HLA-A* and *HLA-B* have similar structural features and present peptides to T cells. While *HLA-C* might be less involved in T cell responses [15], *HLA-C* is responsible for acting as a ligand for natural killer cell receptors, such as killer-cell immunoglobulin-like receptors (KIRs) [16]. *HLA-B* with the Bw4 allotype is a ligand for KIR3DL1. While *HLA-C1* is a ligands for KIR2DL2, KIR2DL3, and KIR2DS2, *HLA-C2* binds to KIR2DL1 and KIR2DS1 [17].

Our previous study showed that *HLA* class I, *HLA-A*, a highly polymorphic gene, varies significantly among different ethnicities and is associated with SARS-CoV-2 severity. We observed that *HLA-A* expression levels showed a significant difference between SARS-CoV-2 severity among symptomatic and asymptomatic individuals (*p* = 0.0005). We also saw a significant difference in *HLA-A* expression levels between South African Black and South African Indian individuals (*p* < 0.0001) [18]. *HLA-B* and *C* were associated with COVID-19 disease in other populations such as Brazilian, Western Indian, Egyptian, Spanish, Asian, European, Chinese, Vietnamese, Taiwanese, Hong Kong Chinese, Italian, and Ecuadorians. Increased *HLA-C* expression was associated with protection against HIV-1 [19]. Some *HLA* alleles have been associated with mild COVID-19 when compared to severe COVID-19 [20]. *HLA-B* is involved in peptide presentation; its alleles have been associated with COVID-19 [6,21] and expression levels in human lung cells of SARS-CoV-2-infected individuals [22]. *HLA-B* expression was also associated with Behçet’s disease [23].

In this paper, we investigated the *HLA-B* and *C* expression levels across South African ethnicities and whether these expression level variations influence COVID-19 severity in a particluar ethnic group. Real-time PCR was used to identify the *HLA* expression levels. To our knowledge, this was the first study to analyze the relationship between *HLA-B* and *HLA-C* mRNA expression levels and COVID-19 severity in different South African ethnicities.

## 2. Methods

In this study, we used a cohort of SARS-CoV-2 positive individuals (SARS-CoV-2 Antibody Prevalence Study (SAP; *n* = 591)). The ethnic groups analyzed were South African Black (*n* = 148) and South African Indian (*n* = 111) individuals. We grouped patients into two groups according to their infection severity, which included South African Black asymptomatic (*n* = 35), South African Black symptomatic (*n* = 113), South African Indian asymptomatic (*n* = 8), and South African Indian symptomatic (*n* = 101). SARS-CoV-2 diagnosis was performed by using the TaqPath COVID-19 RT-PCR kit (Thermo Fisher Scientific, Waltham, MA, USA) and the QuantStudio 5 Real-Time PCR system (Applied Biosystems, Woburn, MA, USA), as per the manufacturer’s directions. This was a longitudinal cohort of SARS-CoV-2-infected individuals. In 2021 and 2022, buffy coat samples (*n* = 560) were gathered at 6 weeks post-infection. SARS-CoV-2-positive nasopharyngeal swabs (*n* = 117) were also obtained. All samples, along with the necessary information such as demographics, clinical data, personal information (age, gender, race), and informed consent, were obtained.

Symptomatic participants presented at least one of the following symptoms: oxygen required, hospitalization, pneumonia, confusion, sore throat, loss of taste/smell, nausea/vomiting, headache, shortness of breath, body aches, fatigue, cough, fever, or chills. Asymptomatic participants did not present any symptoms. We grouped comorbidities into non-communicable (e.g., obesity, anemia, hypertension, cardiovascular disease, asthma, diabetes, and cancer) and communicable (e.g., HIV).

Ethical approval for this study was obtained from the Biomedical Research Ethics Committee (BREC) at the University of KwaZulu-Natal; protocol reference number: BREC/00002648/2021.

To test whether *HLA-B* and *C* mRNA expression levels were associated with COVID-19 and their contributing factors such as age, gender, comorbidities, disease severity, and ethnicity, we examined SARS-CoV-2-infected individuals of different ethnicities recruited from South Africa, in which the estimated effect of each *HLA* allele on COVID-19 and its contributing factors were reported. 

Nucleic acid was extracted from buffy coat samples as per the manufacturer’s directions; the RNA extraction was stored at 20 °C. Thereafter, cDNA was prepared using the iscript cDNA synthesis kit (BioRad, Hercules, CA, USA) according to manufacturer guidelines and then stored at 20 °C. The *HLA-B* and *HLA-C* mRNA expression levels were obtained using a real-time PCR (RT-PCR) protocol (Thermo Fisher Scientific) on the QuantStudio 5 instrument as per the manufacturer’s guidelines. PowerUp SYBR Green Master Mix (ThermoFisher Scientific) was prepared as per manufacturer guidelines, and RT-PCR was performed. RT-PCR cycling conditions and catalog numbers for *HLA-B* and *HLA*-*C* are available on request. The RT-PCR conditions and primers used are as previously published [24]. The B2M housekeeping gene was used in this study. The B2M primer sequence was as follows: forward primer: -GACTTGTCTTTCAGCAAGGA; reverse primer: -ACAAAGTCACATGGTTCACA.

## 3. Statistical and Bioinformatics Analysis

GraphPad Prism 8 software was used for analysis. The unpaired Welch t-test that was two-tailed and a one-way ANOVA *t*-test Bonferroni were used to compare variables; categorical variables were analyzed using Fisher’s exact tests. A *p*-value of less than 0.05 was considered statistically significant.

## 4. Results

### 4.1. HLA-B and C mRNA Expression Levels across Different COVID-19 Disease Severity Levels

**Figure 1:**
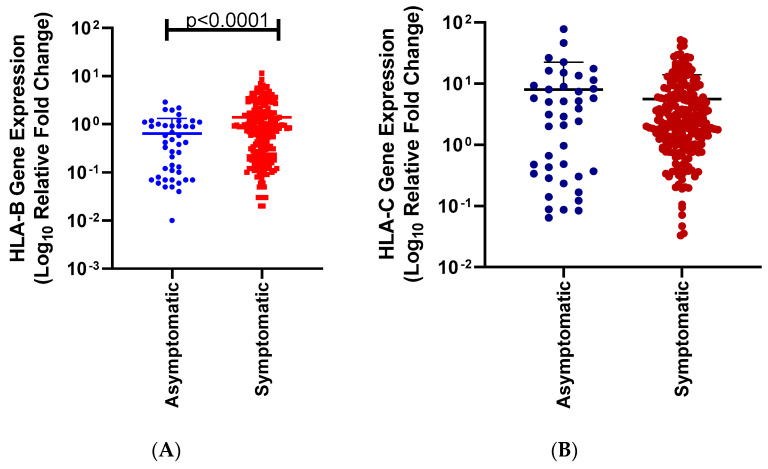
*HLA-B* mRNA expression levels among symptomatic and asymptomatic SARS-CoV-2 infected individuals. There was a significant difference between symptomatic (red squares) and asymptomatic (blue dots) individuals. Asymptomatic individuals *HLA-C* expression levels were significantly lower than symptomatic individuals (*p* = 0.0001, (**A**)). *HLA-C* mRNA expression levels among SARS-CoV-2 infected individuals of different disease severity. There is no significant difference between the *HLA-C* expression levels among asymptomatic (blue) and symptomatic individuals (red) (*p* = 0.2914, (**B**)).

In this study, we investigated the effect of *HLA-B* and *HLA-C* mRNA expression levels on COVID-19 severity in positive COVID-19 individuals. The *HLA-B* mRNA expression level mean and *p*-values are summarized in the text, and *p*-values in the figures. For all individuals, *HLA-B* mRNA expression levels were significantly lower in symptomatic individuals, suggesting protection against COVID-19 (95% CI 0.4463 to 1.052; asymptomatic mean = 0.6389; symptomatic mean = 1.388; *p* = 0.0001, Figure 1). 

### 4.2. HLA-B and HLA-C mRNA Expression Levels across Different Ethnic Groups

**Figure 2:**
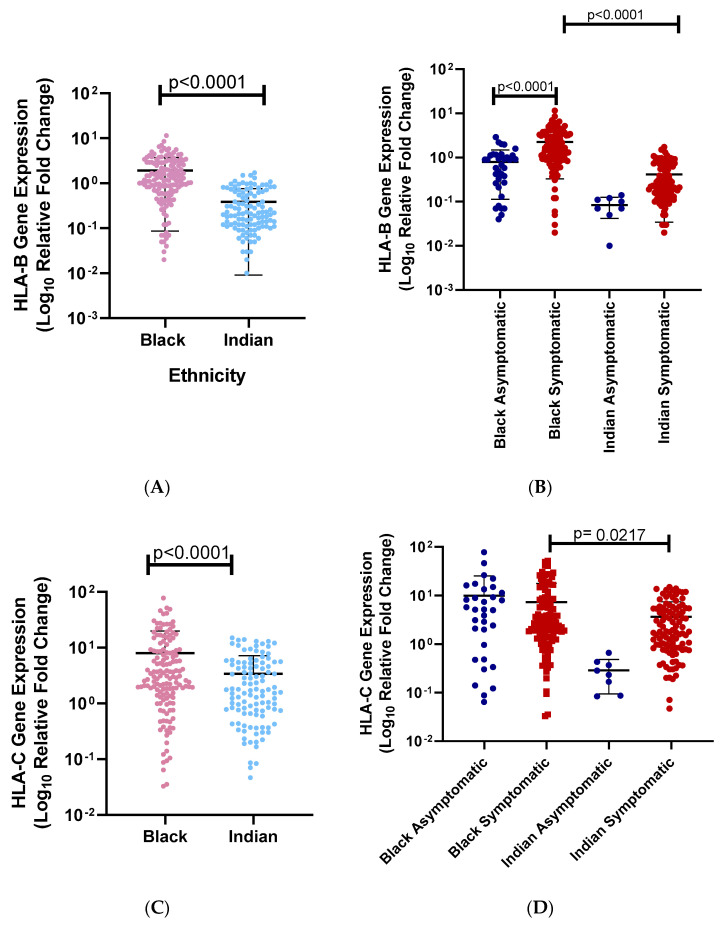
(**A**) *HLA-B* mRNA expression levels among different ethnic groups. We found that South African Black individuals’ *HLA-B* mRNA expression levels are significantly higher than South African Indian individuals (*p* = 0.0001). (**B**) *HLA-B* mRNA expression levels among symptomatic and asymptomatic individuals of South African ethnicity. A comparison between South African Black symptomatic, South African Black asymptomatic, South African Indian symptomatic, and South African Indian asymptomatic individuals. There is a significant association between *HLA-B* in South African Black symptomatic (red dots) and South African Black asymptomatic individuals (blue dots) (*p* < 0.0001). *HLA-B* mRNA expression levels were significantly higher in South African Black symptomatic (red dots) than in South African Indian symptomatic (red dots) individuals (*p* < 0.0001). The *HLA-B* mRNA expression of asymptomatic South African Black (blue dots) individuals was not associated with asymptomatic South African Indian individuals (blue dots) (*p* > 0.9999). Symptomatic South African Indian individuals (red dots) *HLA-B* mRNA expression levels were not significantly associated with asymptomatic South African Indian individuals (blue dots) (*p* > 0.9999). (**C**) *HLA-C* mRNA expression levels among different ethnic groups. We found that South African Black individuals had significantly higher *HLA-C* mRNA expression levels than South African Indian individuals (*p* < 0.0001). (**D**) *HLA-C* mRNA expression levels among symptomatic and asymptomatic individuals from South African ethnicities. A comparison between South African Black symptomatic, South African Black asymptomatic, South African Indian symptomatic, and South African Indian asymptomatic individuals. We did not observe a significant difference between *HLA-C* mRNA expression levels in South African Black symptomatic (red dots) and South African Black asymptomatic individuals (blue dots) (*p* = 0.9443). *HLA-C* mRNA expression levels were significantly higher in South African Black symptomatic (red dots) than in South African Indian symptomatic (red dots) individuals (*p* = 0.0217). The *HLA-C* mRNA expression levels of asymptomatic South African Black (blue dots) individuals were not significantly different from those of asymptomatic South African Indian individuals (blue dots) (*p* = 0.0520). Symptomatic South African Indian individuals’ (red dots) *HLA-C* mRNA expression levels were not significantly different from those of asymptomatic South African Indian individuals (blue dots) (*p* > 0.9999).

We first looked at the relationship between ethnicity and disease severity. We saw an association with disease severity and ethnicity (*p* = 0.0003, Table 1). (A) We then analyzed the relationship between *HLA-B* mRNA expression levels and different ethnic groups. South African Black individuals have significantly higher *HLA-B* mRNA expression levels than South African Indian individuals (95% CI −1.835 to −1.225; South African Black mean = 1.914, South African Indian mean = 0.3837; *p* < 0.0001, Figure 2A). (B) In addition, we look at the *HLA-B* mRNA expression levels between ethnicity and disease severity. There is a significant difference between symptomatic and asymptomatic South African Black individuals (*p* < 0.0001). Similarly, there is a significant difference between symptomatic and asymptomatic South African Indian individuals (*p* < 0.0001). *HLA-B* mRNA expression levels are higher among asymptomatic South African Black than asymptomatic South African Indian individuals, and symptomatic South African Black individuals and symptomatic South African Indian individuals (*p* < 0.0001; *p* < 0.0001), respectively, suggesting significant protection in South African Black individuals (mean asymptomatic South African Black = 0.799; mean symptomatic South African Black = 2.26; mean asymptomatic South African Indian = 0.0838; mean symptomatic South African Indian = 0.414; Figure 2B). (C) We also observed the effect of ethnicity on *HLA-C* mRNA expression levels. South African Black individuals have significantly higher *HLA-C* mRNA expression levels than South African Indian individuals (95% CI −6.636 to −2.497, South African Black mean = 7.969, South African Indian mean = 3.403, *p* = 0.0001, Figure 2C). (D) In addition, we looked at the *HLA-C* mRNA expression levels between ethnicity and disease severity (South African Black asymptomatic mean = 9.99, South African Black symptomatic mean = 7.38, South African Indian asymptomatic mean = 0.290, South African Indian symptomatic mean = 3.65; Figure 2D). *HLA-C* mRNA expression levels were significantly higher in South African Black symptomatic individuals than in South African Indian symptomatic individuals (*p* < 0.0034). The *HLA-C* mRNA expression levels of asymptomatic South African Black individuals were significantly higher than asymptomatic South African Indian individuals (*p* < 0.006). Symptomatic South African Indian *HLA-C* mRNA expression levels were significantly higher than asymptomatic South African Indian individuals (*p* < 0.0001). Disease severity was divided according to ethnicity (Table 1).

### 4.3. HLA-B and HLA-C mRNA Expression Levels across Different Genders

**Figure 3:**
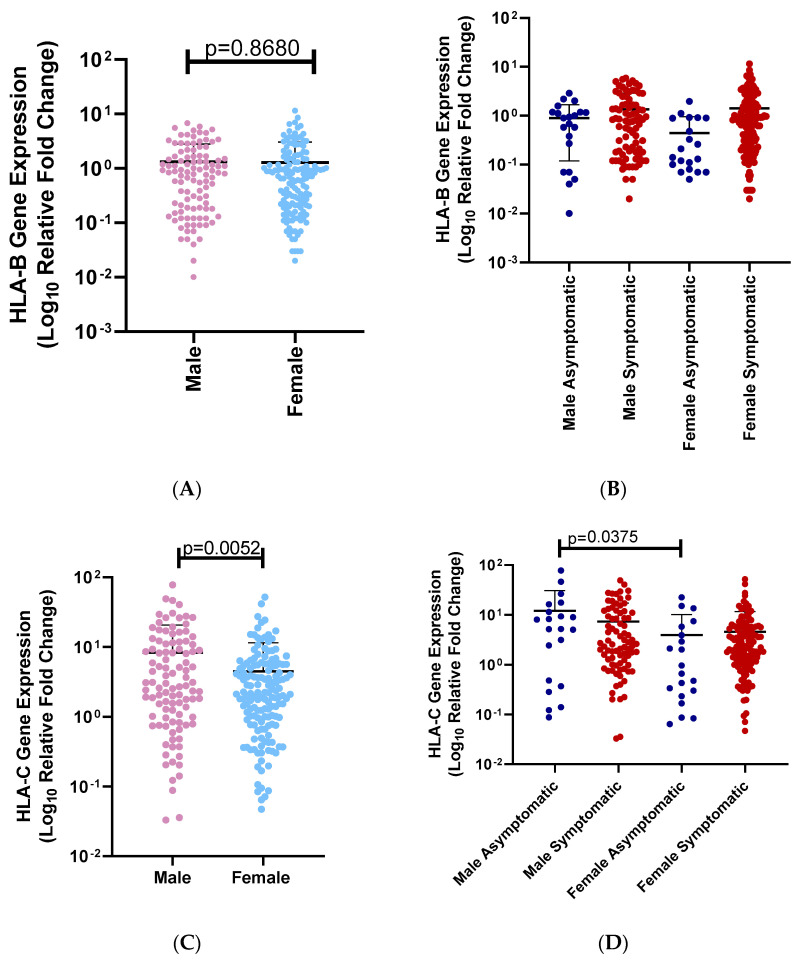
(**A**) *HLA-B* mRNA expression levels of SARS-CoV-2-infected South African individuals among different genders. A comparison of *HLA-B* mRNA expression levels between infected SARS-CoV-2 South Africans and gender. There is no significant difference between *HLA-B* mRNA expression levels in males (light red dots) and females (light blue dots) (*p* = 0.8680). (**B**) *HLA-B* mRNA expression levels among symptomatic and asymptomatic SARS-CoV-2-infected males and females. A comparison between *HLA-B* expression levels among symptomatic males (red dots), symptomatic females (blue dots), asymptomatic males (blue dots), and asymptomatic females (red dots). There is a significant association with *HLA-B* expression levels in females who are symptomatic (red dots) and asymptomatic (blue dots) (*p* = 0.0001). *HLA-B* expression levels was significantly different between male symptomatic (red dots) and asymptomatic males (blue dots) (*p* = 0.2407). There was no significant association with *HLA-B* in males who were symptomatic (red dots) and females who were symptomatic (red dots) (*p* = 0.9944). There is a significant association with *HLA-B* in males who are asymptomatic (blue dots) and females who are asymptomatic (blue dots) (*p* = 0.1390). (**C**) *HLA-C* mRNA expression levels of infected SARS-CoV-2 South African individuals among different genders. A comparison of *HLA-B* mRNA expression levels between infected SARS-CoV-2 South African individuals and gender. There is a significant difference between *HLA-C* mRNA expression levels in males (light red dots) and females (light blue dots) (*p* = 0.0052). (**D**) *HLA-C* mRNA expression levels among symptomatic and asymptomatic SARS-CoV-2-infected males and females. A comparison between *HLA-C* expression levels among symptomatic males (red dots), and females (blue dots), asymptomatic males (blue dots) and females (red dots). There was no significant difference between *HLA-C* expression levels in symptomatic females (red dots) and asymptomatic females (blue dots) (*p* > 0.9999). There was no significant difference between symptomatic males (red dots) and asymptomatic males (blue dots) (*p* = 0.2431). There was no significant association with *HLA-B* expression levels in males who were symptomatic (red dots) and females who were symptomatic (red dots) (*p* = 0.2428). However, there was a significant difference between *HLA-C* expression levels in males who were asymptomatic (blue dots) and asymptomatic females (blue dots) (*p* = 0.0375).

We analyzed the relationship between gender and COVID-19 disease severity. There was no significant association between gender and COVID-19 severity (Table 2, *p* = 0.1088). (A) We then analyzed the effect of gender on *HLA-B* mRNA expression levels. There was no significant difference between *HLA-B* mRNA expression levels of males and females (females means = 1.281, males mean = 1.315; *p* = 0.8680, Figure 3A). (B) We did not observe a significant difference between the *HLA-B* mRNA expression of asymptomatic males and symptomatic males (*p* > 0.9999; Figure 3B). We also did not observe a significant difference between symptomatic males and females (*p* > 0.9999). *HLA-B* mRNA expression levels were not significantly different between asymptomatic males and asymptomatic females (*p* > 0.9999). *HLA-B* mRNA expression levels were not significantly associated with asymptomatic females and symptomatic females (*p* < 0.0749) (male asymptomatic mean = 0.899; male symptomatic mean = 1.35; female asymptomatic = 0.446; female symptomatic = 1.41). (C) We then analyzed the effect of gender on *HLA-C* mRNA expression levels. There was a significant difference between *HLA-C* mRNA expression levels of males and females (females means = 0.04701, males mean = 0.03294; *p* = 0.0052, Figure 3C). (D) We did not observe a significant difference between the *HLA-C* mRNA expression levels of asymptomatic males and symptomatic males (*p* = 0.2431; Figure 3D). We also did not observe a significant difference between symptomatic males and females (*p* = 0.2428). *HLA-C* mRNA expression levels were significantly different between asymptomatic males and asymptomatic females (*p* = 0.0375, Figure 3D). *HLA-C* mRNA expression levels were not significantly different between asymptomatic females and symptomatic females (*p* > 0.9999). (male asymptomatic mean = 12.0; male symptomatic mean = 7.31; female asymptomatic = 3.95; female symptomatic = 4.59). Disease severity was grouped according to gender (Table 2).

### 4.4. HLA-B and HLA-C mRNA Expression Levels across Existing Conditions

**Figure 4:**
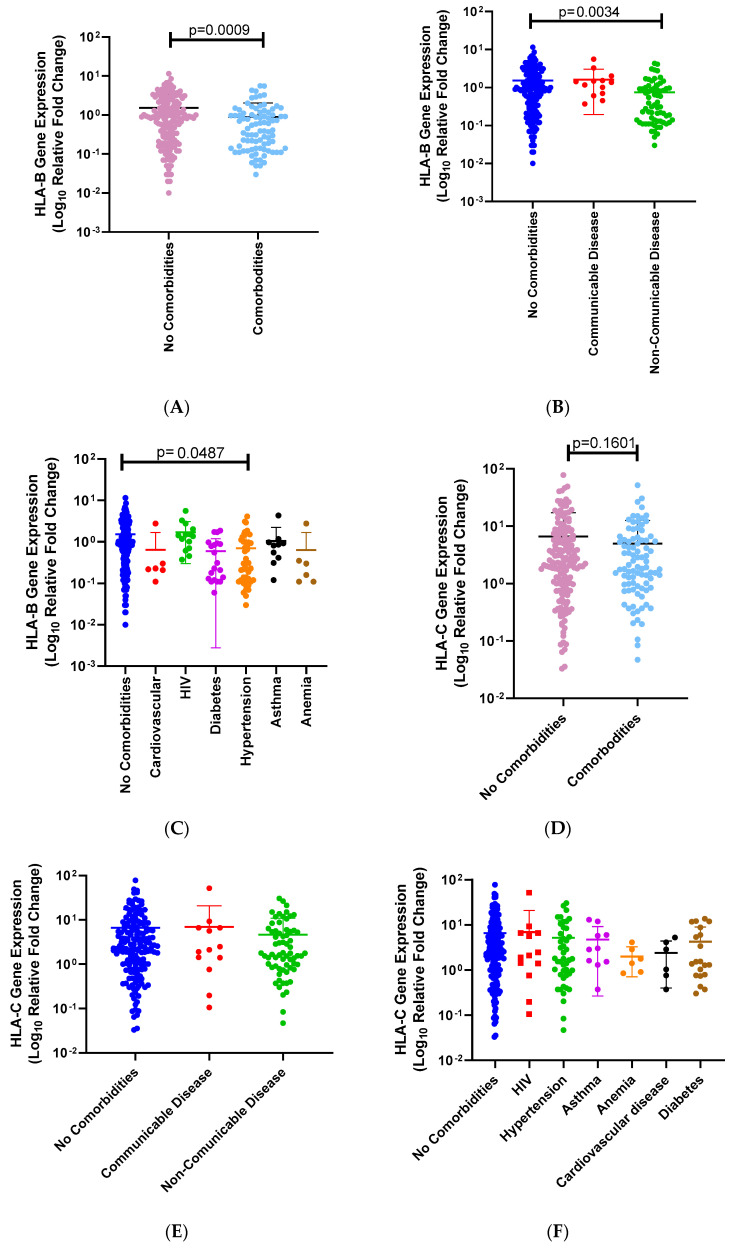
(**A**) *HLA-B* mRNA expression levels and the presence and absence of comorbidities among SARS-CoV-2-infected individuals. We compared *HLA-B* mRNA expression levels among SARS-CoV-2-infected individuals with or without comorbidities. There is a significant association between *HLA-B* mRNA expression levels among SARS-CoV-2-infected individuals with (blue dots) and without comorbidities (red dots) (*p* = 0.0009, (**A**)). (**B**) *HLA-B* mRNA expression levels among SARS-CoV-2-infected individuals with communicable, noncommunicable, or no comorbidities. No comorbidities (blue dots), communicable disease (red dots), or non-communicable disease (green dots). Significant differences were observed between no comorbidities and non-communicable diseases (*p* = 0.0034, (**B**)). Individuals with no comorbidities had significantly higher *HLA-B* mRNA expression levels than those with non-communicable diseases. (**C**) We then analyzed the relationship between *HLA-B* expression levels among different types of comorbidities among SARS-CoV-2-infected individuals. We compared no comorbidities (blue dots) with different types of diseases, such as cardiovascular disease (red dots), HIV (green dots), diabetes (purple dots), hypertension (orange dots), asthma (black dots), and anemia (brown dots). There was only a significant difference between no comorbidities and hypertension (*p* = 0.00487, (**C**)). (**D**) The relationship of comorbidities and *HLA-C* mRNA expression levels. There is a significant association between *HLA-C* mRNA expression levels among SARS-CoV-2 infected individuals with (blue dots) and without comorbidities (light red dots) (95% CI = −3.883 to 0.6446; no comorbidities mean = 6.602; comorbidities mean = 4.983; *p* = 0.1601 (**D**)). (**E**) We then divided the comorbidities into communicable and non-communicable diseases. We observed no significant difference between all three categories (no comorbidities mean = 6.60; communicable disease mean = 6.97; noncommunicable disease = 4.64, (**E**)). (**F**) We further divided the communicable and non-communicable diseases. (no comorbidities mean = 6.60; HIV mean = 6.97; hypertension mean = 5.19; asthma mean = 4.74; anemia mean = 1.99; cardiovascular disease mean = 2.41; diabetes mean = 4.24, (**F**)). There was no significant difference between any of the categories (**F**).

In addition, we analyzed the relationship between COVID-19 severity and comorbidities. There was a significant difference between COVID-19 severity and the presence of comorbidities (Table 3, *p* = 0.0528). (A) We also analyzed the relationship between comorbidities and *HLA-B* mRNA expression levels. There is a significant association between *HLA-B* mRNA expression levels among SARS-CoV-2-infected individuals with and without comorbidities (95% CI = −0.9944 to −0.2584; no comorbidities mean = 1.513, comorbidities mean = 0.8867; *p* = 0.0009; Figure 4A). (B) We then divided the comorbidities into communicable and non-communicable diseases. We observed a significant difference between no comorbidities and non-communicable diseases (*p* = 0.0034; no comorbidities mean = 1.51, communicable disease mean = 1.62, noncommunicable disease = 0.747; Figure 4B). (C) We further divided the communicable and non-communicable diseases (no comorbidities mean = 1.51, HIV mean = 1.70, hypertension mean = 0.703, asthma mean = 1.04, anemia mean = 0.632, cardiovascular disease mean = 0.638, diabetes mean = 0.597; Figure 4C). There was a significant difference between no comorbidities and hypertension (*p* = 0.0487; Figure 4C). (D) In addition, we analyzed the relationship of comorbidities and *HLA-C* mRNA expression levels. There was a significant association between *HLA-C* mRNA expression levels among SARS-CoV-2-infected individuals with and without comorbidities (95% CI = −3.883 to 0.6446; no comorbidities mean = 6.602, comorbidities mean = 4.983; *p* = 0.1601; Figure 4D). (E) We then divided the comorbidities into communicable and non-communicable diseases. We observed no significant difference between all three categories (*p* = 0.9999, *p* = 0.4964, *p* = 0.9999; no comorbidities mean = 6.60, communicable disease mean = 6.97, noncommunicable disease = 4.64, Figure 4E). (F) We further divided the communicable and non-communicable diseases (no comorbidities mean = 6.60; HIV mean = 6.97; hypertension mean = 5.19; asthma mean = 4.74; anemia mean = 1.99; cardiovascular disease mean = 2.41; diabetes mean = 4.24; Figure 4F). There was no significant difference between any of the categories (Figure 4F). Asymptomatic and symptomatic were separated into no comorbidities and comorbidities (Table 3).

### 4.5. HLA-B and C mRNA Expression Levels across Different Age Groups

**Figure 5:**
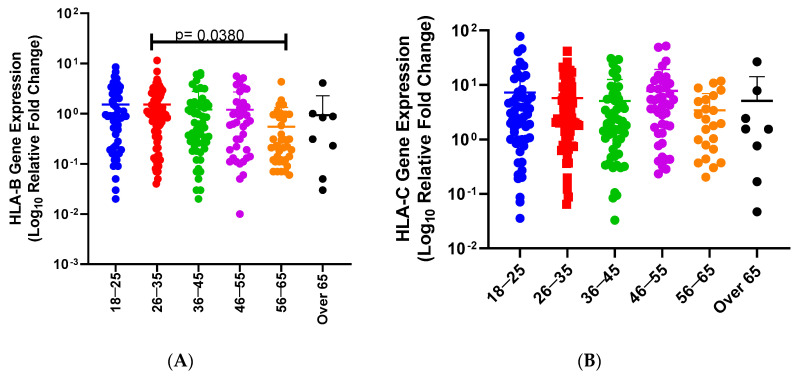
*HLA-B* mRNA expression levels and age among SARS-CoV-2-infected individuals in South Africa. A comparison between *HLA-B* expression levels among SARS-CoV-2 infected individuals and age (18–25 (blue dots), 26–35 (red dots), 36–45 (green dots), 46–55 (purple dots), 56–65 (orange dots), and >65 (black dots)). We also observed a significant difference between 26–35 and 56–65 years (*p* = 0.0380, (**A**)). There is no significant association between *HLA-B* mRNA expression levels among SARS-CoV-2-infected individuals and any of the other age groups. (**B**) *HLA-C* mRNA expression levels and age among SARS-CoV-2-infected individuals. A comparison between *HLA-C* mRNA expression levels among SARS-CoV-2-infected individuals and age (18–25 (blue dots), 26–35 (red squares), 36–45 (green dots), 46–55 (purple dots), 56–65 (orange dots), and >65 (black dots)). There is no significant association between *HLA-C* mRNA expression levels among SARS-CoV-2-infected individuals and any of the age groups (**B**).

Finally, we examined the relationship between age and disease severity. We did not find an association between age and disease severity (*p* = 0.6709). We then determined the effect of age groups on *HLA-A* mRNA expression levels in SARS-CoV-2-infected individuals (Figure 5). We determined the effect of age groups on *HLA-B* mRNA expression levels in SARS-CoV-2-infected individuals (18–25 mean = 1.53; 26–35 mean = 1.52; 36–45 mean = 1.21; 46–55 mean = 1.20; 56–65 mean = 0.549; over 65 mean = 0.930; Figure 5A). Previously, COVID-19 disease was associated with age. We also observed a significant difference between 26–35 and 56–65 years (*p* = 0.0380). We determined the effect of age groups on *HLA-C* mRNA expression levels in SARS-CoV-2-infected individuals (18–25 mean = 7.28; 26–35 mean = 5.74; 36–45 mean = 5.08; 46–55 mean = 7.76; 56–65 mean = 3.43; over 65 mean = 5.12; Figure 5B) Previously, COVID-19 disease was associated with age. We did not observe a significant difference between any of the age groups and *HLA-C* mRNA expression levels. Individuals of a particular age group were divided according to disease severity levels (Table 4).

## 5. Discussion

We aimed to determine the effects of *HLA-C* and *HLA-B* mRNA expression levels on disease severity, age, ethnic groups, gender, and comorbidities in SARS-CoV-2-infected individuals. We compared *HLA-B* and *HLA-C* mRNA expression levels within the South African SARS-CoV-2 cohort.

Previous studies have shown that disease severity varies across ethnicities. There have been less severe outcomes among the African populations, and COVID-19 has been less deadly in Africa compared to other continents [25]. We observed that *HLA-B* and *HLA-C* mRNA expression levels were both significantly higher in South African Black individuals than in South African Indian individuals (*p* < 0.0001).

Various studies have analyzed the association of *HLA-B* and *HLA-C* with other infectious diseases [19,23]. These studies have confirmed the importance of *HLA* alleles in disease severity. These results strengthen the significance of the role of *HLA* in COVID-19. In this study, we observed that symptomatic COVID-19-infected individuals had significantly higher *HLA-B* mRNA expression than asymptomatic individuals. However, *HLA-C* mRNA expression levels showed no significant difference between symptomatic and asymptomatic individuals. This suggests that *HLA-B* mRNA expression levels are associated with increased disease severity, while *HLA-C* expression might not play a significant role in COVID-19 disease severity. Furthermore, we showed a significant difference in *HLA-B* mRNA expression levels among South African Black asymptomatic and symptomatic individuals (*p* < 0.0001). In addition, symptomatic South African Indian individuals and South African Black individuals also showed a significant difference (*p* > 0.0001). These results suggest that *HLA-B* is associated with worse disease outcomes. *HLA-C* was not significantly different between symptomatic South African Black individuals and asymptomatic South African Black individuals and between symptomatic South African Indian individuals and asymptomatic South African Indian individuals. *HLA-C* mRNA expression levels were significantly higher in symptomatic South African Black individuals than in symptomatic South African Indian individuals (*p* > 0.0217). Some of these findings are consistent with studies that confirm an association between disease severity and ethnicity [26]. 

Males had significantly higher *HLA-C* mRNA expression levels compared to females (*p* = 0.0052). However, there was no significant difference between the *HLA-B* mRNA expression levels and gender (*p* = 0.8680). More studies are required to determine the effect of gender on COVID-19, if any. We did not observe any significant difference between genders with varying infectious states and *HLA-B* expression. There was a significant difference between *HLA-C* expression levels among asymptomatic males and asymptomatic females (*p* = 0.0375). Naemi et al. showed that there was no relationship between gender and COVID-19 severity in the South Asian population [27].

We observed that *HLA-B* mRNA expression levels were significantly higher in individuals who had no comorbidities than those with comorbidities (*p* = 0.0009). We did not observe a significant difference between comorbidities and no comorbidities for *HLA-C* mRNA expression levels (*p* = 0.1601). We further divided comorbidities and did not see a difference between the different comorbidities. *HLA-B* mRNA expression was significantly different between noncommunicable diseases and no comorbidities (*p* = 0.0034). However, there is no significant difference between comorbidities and communicable or non-communicable diseases for *HLA-C* mRNA expression levels. Comorbidities contribute to the disease state and are associated with *HLA-B* mRNA expression levels. However, *HLA-B* mRNA expression levels were significantly higher in individuals with no comorbidities than hypertension (*p* = 0.0487). This suggests that *HLA-B* mRNA expression levels have a relationship with hypertension in SARS-CoV-2-infected individuals. SARS-CoV-2-infected individuals over 65, predominantly males, with comorbidities or organ-associated pathologies are at increased risk of developing severe, critical COVID-19 and death [28]. Type I diabetes mellitus was associated with a severe SARS-CoV-2 infection. Hypertension and cardiovascular disease are also increasing the risk of worse COVID-19 [29]. 

Increased age has been previously associated with increased or worse disease progression; however, we did not see an association between age and COVID-19 severity. Our results showed that *HLA-B* mRNA expression levels among ages 26–35 were significantly higher than 56–65 (*p* = 0.0380). However, there was no significant association between age and *HLA-C* mRNA expression levels in SARS-CoV-2-infected individuals. Surprisingly, *HLA-B* mRNA expression was higher in younger individuals than older individuals; therefore, *HLA-B* might play a role in COVID-19 disease severity among younger individuals. There are controversial results on the effect of age on *HLA* alleles. Izaks et al. did not find an association between *HLA* and death after 85 years. Another study showed that *HLA* class I expression was reduced in lymphocytes and monocytes of the elderly [30]. The *HLA* mRNA level was higher in PBL for *HLA-B* than *HLA-A*. This suggests *HLA* class-I molecules expressed on the cell surface are associated with specific mRNA levels. *HLA* class-I locus expression is cell type-dependent. This cellular differential expression of *HLA* class-I loci could act on the immune response by preferentially presenting distinct peptides to T cells. There was a significant decrease in the amounts of *HLA-A* and *HLA-B* transcripts with increasing age [31]. In a South Asian population, patients who were admitted into the ICU were significantly older than those with mild COVID-19 (*p* < 0.001) [27]. 

Augusto et al. found that HLA-B*15 has been associated with an asymptomatic SARS-CoV-2 infection [32]. HLA-B expression levels have been associated with SARS-CoV-2 symptoms, while HLA-C expression levels were not associated with SARS-CoV-2 symptoms. Increased HLA-B expression was associated with symptomatic individuals. More research within an African population is required to unravel the role of *HLA* expression level on COVID-19 symptoms and other factors and diseases. Researchers should include individuals of different ethnicities and a large sample size. The limiting factor of this study was the sample size of the asymptomatic individuals. In addition, the age groups were limited in this study. Genetic studies are important to develop specific therapeutics to alleviate disease among specific populations. *HLA* studies are significant because they are involved in host responses to pathogens. 

## 6. Conclusions

In Africa, the low COVID-19 mortality rate compared to the rest of the world came as a surprise. This might be attributed to human genetics and other contributing factors discussed in this paper. The reason behind the effect of COVID-19 on Africa is yet to be unraveled. We showed that *HLA* expression levels differ among different South African ethnic groups. In addition, comorbidities are associated with COVID-19 and *HLA-B* expression levels. This is evidence that COVID-19 disease severity is dependent on host genetics.

## Figures and Tables

**Table 1:** The frequency of South African Black and Indian individuals and disease severity.

Ethnicity	Asymptomatic	Symptomatic	Total	*p*-Value
South African Black	35	113	148	
South African Indian	8	101	109	
Total	43	214	257	0.0003

There was a significant difference between ethnicity and disease severity (*p* = 0.0003).

**Table 2:** The frequency of males and females that are asymptomatic and symptomatic.

Gender	Asymptomatic	Symptomatic	Total	*p*-Value
Male	21	82	103	
Female	20	128	116	
Total	41	210	251	0.1088

There was no significant difference between disease severity and gender (*p* = 0.1088).

**Table 3:** The frequency of individuals who did or did not present with comorbidities that are asymptomatic or symptomatic.

Comorbidities	Asymptomatic	Symptomatic	Total	*p*-Value
Not present	34	138	172	
Present	8	72	80	
Total	42	210	252	0.0528

There was no significant difference between asymptomatic and symptomatic individuals who did and did not present with comorbidities (*p* = 0.0528).

**Table 4:** The frequency of individuals who are under and over 45 years old that are asymptomatic and symptomatic.

Age (Years)	Asymptomatic	Symptomatic	Total	*p*-Value
Under 45	31	160	191	
Over 45	10	61	71	
Total	41	221	262	0.6709

There is no significant difference between disease severity and ages below and above 45 (*p* = 0.6709).

## Data Availability

The raw data supporting the conclusions of this article will be made available by the authors on request.
